# Antimicrobial and Osteogenic Effects of Collagen Membrane Decorated with Chitosan–Nano-Hydroxyapatite

**DOI:** 10.3390/biom13040579

**Published:** 2023-03-23

**Authors:** Milos Lazarevic, Sanja Petrovic, Tania Vanessa Pierfelice, Nenad Ignjatovic, Adriano Piattelli, Tamara Vlajic Tovilovic, Milena Radunovic

**Affiliations:** 1 School of Dental Medicine, University of Belgrade, 11 070 Belgrade, Serbia; 2 Department of Medical, Oral and Biotechnological Sciences, University G. d’Annunzio of Chieti-Pescara, 66100 Chieti, Italy; 3 Institute of Technical Sciences of the Serbian Academy of Sciences and Arts, 11 070 Belgrade, Serbia; 4 School of Dentistry, Saint Camillus International University of Health and Medical Sciences, 00131 Rome, Italy; 5 Facultad de Medicina, UCAM Universidad Catolica San Antonio de Murcia, 30107 Guadalupe, Spain

**Keywords:** collagen membrane, chitosan, nano-hydroxyapatite, dental pulp stem cells, antimicrobial effect, osteogenic effect

## Abstract

Collagen membranes are routinely used in oral surgery for bone regeneration. Despite their numerous advantages, such as stimulating bone growth, bacterial contamination still remains one of the disadvantages of membrane use. Thus, we assessed the biocompatibility and osteogenic and antibacterial properties of a collagen membrane (OsteoBiol) modified with chitosan (CHI) and hydroxyapatite nanoparticles (HApNPs). Attenuated total reflectance-Fourier transform infrared spectroscopy (ATR FT-IR), X-ray powder diffraction (XRD), and field emission scanning electron microscopy (FE-SEM) were performed for membrane characterization. Biocompatibility was assessed on dental pulp stem cells (DPSCs) by an MTT assay, while the osteogenic effect was assessed by an ALP activity assay and qPCR analysis of osteogenic markers (BMP4, ALP, RUNX2, and OCN). Antimicrobial properties were investigated by counting colony-forming units (CFUs) of *Streptococcus mitis, Porphyromonas gingivalis*, and *Fusobaterium nucleatum* on membranes and in the surrounding medium. Membranes showed no cytotoxicity. ALP activity was higher and ALP, BMP4, and OCN genes were up-regulated in DPSCs on modified membranes compared to unmodified membranes. The CFUs were reduced on modified membranes and in the medium. Modified membranes showed great biocompatibility and a high osteoinductive effect. Additionally, they showed antimicrobial and antibiofilm effects against periopathogens. It can be concluded that the incorporation of CHI and hydroxyapatite nanoparticles in collagen membranes may be advantageous to promote osteogenesis and reduce bacterial adhesion.

## 1. Introduction

Guided bone regeneration (GBR) is a widely used surgical regenerative technique based on the idea of separating bone from soft tissue during the regeneration process. This concept uses barrier membranes as shields, which prevent the migration of the soft tissue inside the defect, allowing the bone to proliferate freely [1]. Resorbable barrier membranes made of collagen are extensively utilized in oral and periodontal surgery [2]. In addition to their role as a mechanical barrier, which also stabilizes the blood cloth and grafting material in the growing bone, collagen membranes also have low immunogenicity, high biocompatibility, and bioactive properties, i.e., they are chemotactic to the periodontal ligament/gingival fibroblasts as well as adhesive to osteoblasts [2,3,4].

The primary shortcoming of collagen membranes is a high resorption rate, which means the membrane is not always present during the whole process of bone formation. This could be additionally accelerated by bacterial contamination (due to the presence of bacterial products that induce collagen degradation) [5,6]. This is especially important since collagen membranes and their surrounding tissue serve as a favorable niche for microbial colonization and infection [7]. Infection of the membrane is a common complication of membrane exposure to the oral cavity, which occurs during the postoperative period. The exposure rate of collagen membranes during the postoperative healing period can be as high as 28% [8]. An infection of the membrane can further lead to the failure of the surgical intervention outcome. For example, Chaushu et al. showed that complete graft augmentation failure occurred in 17% of cases due to membrane exposure and consequent infection [9]. Becker et al. showed that membrane exposure resulted in reduced bone fill after periodontal surgery [10]. In order to prevent infection, antibiotics are frequently administered after GBR. Prophylactic antibiotic use has various shortcomings. The uncontrolled and frequent use of antibiotics could lead to antimicrobial resistance (AMR), and there is also no evidence that the clinical outcomes of GBR are improved if antibiotics are administered [11]. In order to evade systemic antibiotic application, collagen membranes could be modified by incorporating antibacterial substances. These modifications were primarily focused on the incorporation of antibiotics [12,13,14], but attention was also paid to other antimicrobials, such as chitosan, Ag nanoparticles, curcumin, and acetylsalicylic acid [15,16,17].

Recently, chitosan (CHI), an amino polysaccharide, has been the focus of scientific research. It is a type of chitin that has been partly deacetylated and is recognized for its biocompatibility, low immunogenicity, biodegradability, and antibacterial activity [18]. Attempts have also been made to create chitosan-based barrier membranes, which in preclinical trials demonstrated the aforementioned favorable properties but still retained poor mechanical properties [19]. The combination of collagen–CHI barrier membranes presents a promising solution for overcoming these deficiencies [20].

An important characteristic of barrier membranes is the induction of bone formation. As previously mentioned, the adhesion of osteoblasts to collagen membranes has been demonstrated [21]. Additionally, modifications of collagen membranes to enhance the promotion of bone formation have been performed. Some of these modifications include the incorporation of growth factors [22,23], plant extracts [24] or hydroxyapatite [25]. The HAp collagen system is a promising combination since collagen and HAp are the major organic and inorganic components of bone [25]. The exceptional biocompatibility of HAp has been well recognized [26], as has its ability to build complex biocomposite structures that enhance bone regeneration [27]. Additionally, HAp or nano-HAp (HApNPs) particles can be applied to materials of different compositions in order to achieve better biological properties of the same, but microdimensions [28]. This enables the use of HAp in a variety of medical applications, including coatings or decorations on medical implants to promote osteointegration [29]. Recently, several designs of these components have been tested, each of which has shown encouraging outcomes: in periodontal surgery, HAp-collagen membranes have been used in this manner with great success, enhancing osteoinductivity and osteoconduction [30]. On the other hand, CHI–HAp has been shown to be an effective scaffold for the regeneration of bone tissue [31,32,33,34]. To the best of our knowledge, there is no literature about the osteogenic potential of a CHI–HAp collagen membrane on dental pulp stem cells (DPSCs). Furthermore, studies on collagen CHI–HAp membranes did not evaluate their antibacterial or antibiofilm properties [31,35].

The objective of this study was to investigate the biological properties of commercial collagen membranes, which are, for the purpose of this study, decorated with HApNPs and CHI. Specifically, the objectives were to investigate their biocompatibility, osteogenic properties on DPSCs, and antibiofilm activity against monomicrobial biofilms of three bacterial species: *Streptococcus mitis*, *Porphyromonas gingivalis*, and *Fusobacterium nucleatum*.

## 2. Materials and Methods

### 2.1. Materials

Collagen membranes (OsteoBiol Derma, Tecnoss, Giaveno, Italy), derived from porcine dermis after removal of the epithelial layer, were a gift of TecnossVR Dental s.r.l., Torino, Italy. The thickness of the membranes was 0.9 mm (±0.1 mm), as described by the manufacturer [36]. Original membranes were cut into smaller squares to avoid damage and loss of material.

Membrane samples (5 mm × 5 mm) were divided into two groups. The control group consisted of commercial collagen membranes without decoration, while in the test group, collagen membranes were decorated with HAp and CHI (as follows).

An aqueous calcium nitrate (Ca(NO_3_)_2_) solution (150 mL; 26.6 wt.%) was added to a solution of ammonium phosphate ((NH_4_)_3_PO_4_) (7 mL H_3_PO_4_ + 165 mL NH_4_OH + 228 mL H_2_O) at 50 °C over the period of 60 min, while stirring at the rate of 100 rpm. The solution was then subjected to a heat treatment at 100 °C for 60 min [37]. The resulting gel was dried at room temperature in a vacuum dryer for 72 h, after which the final product, HAp powder, was obtained. X-ray diffraction run on the HAp powder confirmed its crystalline nature.

Chitosan with a low molecular weight (P448869 Sigma-Aldrich, St. Louis, MI, USA), dissolved in acetic acid (1 wt.%), was mixed with an ethanol solution of HAp (1.75 wt.%) in a CHI:HAp 1:1 weight ratio, while stirring with a magnetic stirrer at 400 rpm. The membranes (20 × 30 mm lateral dimensions), using a square grid, were cut with a cutter into square pieces (5 × 5 mm lateral dimensions). The samples were covered with 40 µL (using an Acura manual 825 micropipette) of the 1.75 wt.% suspension of CHI and HAp. The covering was carried out by dropping 40 µL of suspension on the surface of the membrane. The membrane modification method has already been applied in our earlier research [38], in which the membrane was decorated with graphene oxide in the same way. 40 µl of suspension is a small amount (one drop) that is finely distributed on the surface of the membrane (5 × 5 mm). The time between dropping and lyophilization was 15 minutes. During 15 min after dropping, no overflow of the suspension over the ends of the membrane was observed; the suspension is adsorbed on the surface.

The liquid phase was left to lyophilize (Freeze Dryer, Christ Alpha 1–2/ LD Plus) at temperatures ranging from −10 to −60 °C and pressures ranging from 0.37 mbar to 0.1 mbar for 5 h. Since no evidence of CHI–HAp leakage was observed during the drying of the coated membranes, it can be concluded that all of the CHI–HAp added to the membrane was adsorbed onto the membrane. The obtained collagen membranes (OsteoBiol) decorated with HAp and CHI were marked as CHI–HAp–collagen.

### 2.2. Material Characterization

The HAp powder and the obtained CHI–HAp–collagen membrane were characterized using X–ray powder diffraction (XRD; Philips PW1050 diffractometer with CuKα1,2 radiation). Field emission scanning electron microscopy (FE-SEM) was performed on a Carl Zeiss ULTRA Plus microscope. Infrared spectroscopy (ATR FT-IR) was carried out on a Nicolet iS10 FT-IR Spectrometer (Thermo Scientific Instruments, Waltham, MA, USA) in the spectral range from 400 to 4000 cm^−1^.

### 2.3. Samples Sterilization

Prior to experiments, all samples were UV irradiated for 30 min per side [38]. Three random samples from both groups were chosen to verify the sterilization process.

### 2.4. Isolation, Cultivation, and Characterization of Dental Pulp Stem Cells (DPSCs)

For the purpose of isolating DPSCs, three semi-impacted wisdom teeth from three healthy patients (22, 23, and 24 years) were included. After obtaining written informed consent, atraumatic tooth extraction was carried out at the Clinic for Oral Surgery, School of Dental Medicine, University of Belgrade, Belgrade, Serbia. The Ethics Committee of the School of Dental Medicine gave the study their seal of approval (Protocol number 36/2). Teeth were delivered immediately to the laboratory, where they underwent additional sterile processing. Phosphate-buffered saline (PBS) solution (Thermo Fisher Scientific, Waltham, MA, USA) was used to properly clean the surfaces of the teeth before DPSCs were isolated and characterized as previously described [39]. In brief, the dental pulp was removed using an endodontic file after the pulp chamber was exposed by crushing the tooth with a sterile clamp. To culture the tissues, they were separated into 1 mm^3^ pieces and put into Dulbecco’s modified Eagle medium with 10% fetal bovine serum and 1% antibiotic-antimycotic solution (all from Thermo Fisher Scientific, Waltham, MA, USA). The cells were kept at 37 °C in a humidified environment that contained 5% CO_2_. Every two to three days, the culture media was replaced. After cell cultures reached 80% confluence, they were passaged. The 5th-passage cells were used for the experiments. The cell experiments were carried out in triplicate and repeated twice.

### 2.5. MTT Assay

Membranes were placed in a 96-well plate by sterile tweezers and DPSCs were seeded (10^4^ cells per well) on top of membranes to test them for cytotoxicity. The cells were then incubated at 37 °C in a humidified 5% CO_2_ environment. After 24, 48 and 72 h, the cell cultures’ incubation was halted by discarding the used medium. Each membrane was transferred to the new 96-well plate and each well was filled with complete medium containing 3-(4,5-dimethylthiazol-2-yl)-2,5-diphenyl tetrazolium bromide (MTT, 0.5 mg/mL) (from Sigma-Aldrich, St. Louis, MI, USA) and incubated for an additional 4 h [39]. After discarding the supernatant, the formazan crystals were broken down in 100 μL of dimethyl sulfoxide (Sigma-Aldrich, St. Louis, MI, USA) by vigorous shaking for 15 min at 37 °C. Utilizing an enzyme-linked immunosorbent assay microplate reader, optical density (OD) was determined at 540 nm (RT-2100c, Rayto, Shenzhen, China). The cell viability (%) was calculated using the formula: ODsample/ODblank × 100 [40].

### 2.6. Alkaline Phosphatase (ALP) Activity Assay

Utilizing the pNPP Alkaline Phosphatase assay kit (Sigma-Aldrich, St. Louis, MI, USA), the alkaline phosphatase (ALP) activity was measured 7, 14, and 21 days after osteogenic differentiation. Briefly, the medium was withdrawn, each membrane was transferred to the new plate and the cell layers were washed three times with PBS and permeabilized overnight at 4 °C with 0.1% Triton X-100 at designated times post-induction. The following day, 50 μL of lysate was added to a 1 M diethanolamine buffer (pH 9.8, containing 0.5 mM MgCl_2_) containing 1 mg/mL pNPP (4-nitrophenyl phosphate disodium salt hexahydrate). In each well, one hundred microliters of pNPP substrate were introduced. The plates were incubated for 30 min at room temperature in order to produce the yellow, water-soluble reaction product. The reaction was terminated by the addition of 3 M NaOH, and the absorbance at 405 nm was measured using an ELISA microplate reader (RT-2100c, Rayto, China). Each well’s ALP activity was adjusted to its total protein content. BioSpec-nano (Shimadzu, Kyoto, Japan) was used to measure the total protein content.

### 2.7. DPSCs Osteodifferentiation

Cells (10^5^ per well in a 24-well plate) were grown for 7, 14, and 21 days in medium for osteogenic differentiation (StemMACS, Miltenyi Biotec, San Francisco, CA, USA) with membranes, with the medium being changed every 2–3 days. After each period of time, membranes were transferred to the new plate and TRIzol was used to extract total RNA (Invitrogen, Thermo Fisher Scientific, Waltham, MA, USA). Complementary DNA was created following the manufacturer’s instructions with the Revert Aid First Strand cDNA Synthesis kit (Thermo Fisher Scientific). Following that, qPCR analysis was carried out on the Line Gene-K Fluorescence Real-time PCR Detection System (Bioer, Hangzhou, China) with the Maxima SYBR Green/ROX qPCR Master Mix (Thermo Fisher, Scientific Waltham, MA, USA). Table 1 shows the sequences of the human-specific primers used.

### 2.8. Antibiofilm Effect

The antibiofilm effect was assessed in vitro by cultivating monomicrobial biofilms on the control and modified membranes. After biofilm formation, quantification of biofilms was performed by counting their colony-forming units (CFUs). In order to assess whether active components (HAp and CHI) were released from the collagen membrane, the CFUs in the medium around specimens were quantified. All analyses were performed in triplicate.

#### 2.8.1. Bacteria Strains and Conditions of Growth

The reference strains *P. gingivalis* ATCC 33277, *F. nucleatum* ATCC 25,586, and *Streptococcus mitis* ATCC 13,770 (Microbiologics KWIK-STIK, Manassas, VA, USA) were used for monomicrobial biofilm formation. The first step in this process is the activation of the reference strains, which was performed under the conditions listed in Table 2.

#### 2.8.2. Biofilm Formation

After activation, a few colonies of each bacterial species were transferred to a suitable medium (Table 2). Bacterial suspensions of each species were centrifuged (10 min, 3000 rpm), the supernatant was discarded, and the pellet was resuspended in PBS (1.0 McFarland standard ≈ 10^8^ cells/mL) (DEN-1 densitometer, Biosan, Riga, Latvia). The final value of CFU/mL of around 10^5^ was obtained by further dilutions in the suitable medium (Table 2).

Sterile collagen membranes (5 mm × 5 mm) were placed with sterile tweezers in a sterile 96-well dish. In order to form a primary pellicle and mimic the conditions of biofilm formation in vivo, collagen membranes were first embedded in 100 μL of artificial saliva (Pharmacy Belgrade, Belgrade, Serbia) for 4 h at 37 °C. This step was followed by the addition of 200 μL of the previously prepared bacterial suspension (around 10^5^ CFU/mL). Incubation was performed in static conditions, as listed in Table 2.

#### 2.8.3. Determination of CFUs of Biofilms Formed on Membranes

The quantification of biofilms formed on membranes was performed by counting CFUs on each membrane. Since biofilm is firmly attached to the surface of the membranes, membranes with biofilms were gently rinsed with PBS in order to detach non-adherent bacterial cells. Then, membranes were transferred to plastic tubes containing 1 mL of sterile PBS and shaken using a thermoshaker (900 rpm, 37 °C). Serial 10-fold dilutions were seeded on agar plates (Table 2). After incubation for 5 days (*P. gingivalis*, *F. nucleatum*) and 48 h (*S. mitis*), colonies were counted.

#### 2.8.4. Determination of CFUs in Medium Surrounding Membranes

This analysis was performed in order to determine whether antibiofilm substances are released from the membranes. A total volume of 20 µL of the medium in which each membrane was incubated for 24 h (in order to form biofilm) was collected. Twelve tenfold serial dilutions of each collected medium were seeded on agar plates. After incubation, colonies were counted.

#### 2.8.5. Scanning Electron Microscopy (SEM) for Monomicrobial *S. mitis* Biofilm Visualization

For visualization of the formed monomicrobial biofilm of *Streptococcus mitis*, scanning electron microscopy was performed. Membranes were removed from the medium and gently rinsed with PBS in order to detach non-adherent bacteria. Samples were fixed in glutaraldehyde (48 h) and then dehydrated by applying a series of solutions of 3% acetic acid, 3% acetic acid and 25% ethanol, 3% acetic acid and 50% ethanol, and 70% ethanol, according to a previously described fixation method [41]. Samples were kept in each solution for 15 min and finally stored in 70% ethanol. Prior to SEM visualization, samples were dried at room temperature for 24 h, coated with a thin gold layer (Polaron SC503, Fisons Instruments) and visualized with SEM. SEM analysis was performed using a TESCAN FESEM (Mira 3 XMU, TESCAN a.s., Brno, Czech Republic) operating at 10 kV.

### 2.9. Statistical Analysis

Analyses were performed by the software package GraphPad Prism ver. 9 (GraphPad Software, Inc.). Independent-sample Student’s t-tests were used after the distribution was checked for normality using the Kolmogorov–Smirnov normality test. The data are presented as mean with SD. The statistical significance level was set at *p* < 0.05.

## 3. Results

### 3.1. Characterization of HAp and CHI–HAp–Collagen Membrane

**Figure 1:**
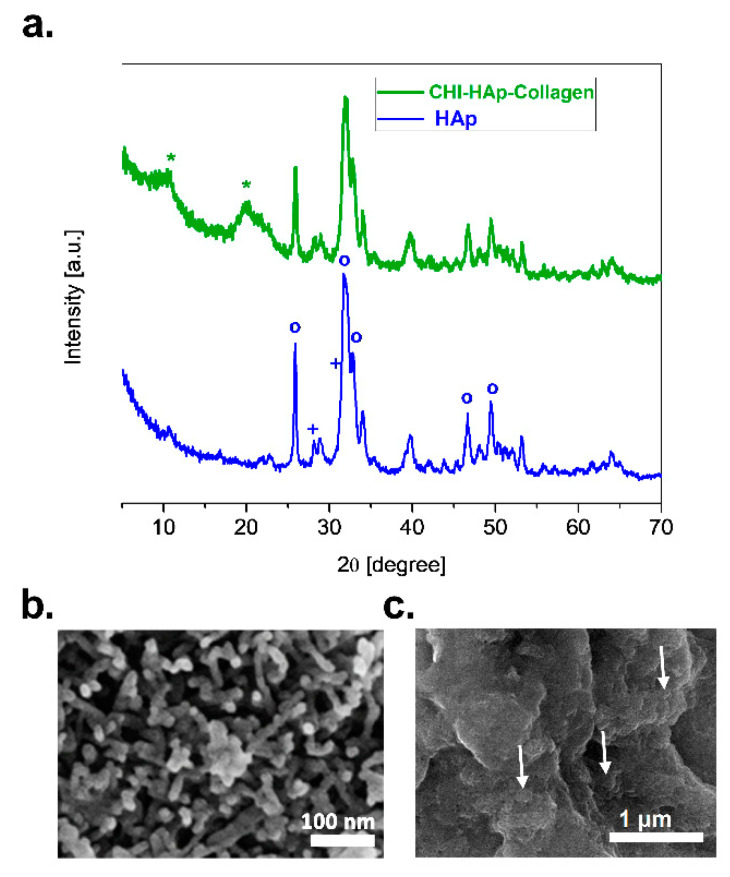
The analysis of (**a**) XRD patterns of HAp powder (O—HAp, +—TCP) and CHI–HAp–collagen (*—CHI). (**b**) FE-SEM of HAp powder and (**c**) FE-SEM of CHI–HAp–collagen (arrows show HApNPs).

The synthesized HAp powder and CHI–HAp–collagen were characterized by X-ray diffraction (XRD) and field emission scanning electron microscopy (FE-SEM) (Figure 1). XRD analysis confirmed the qualitative presence of hydroxyapatite phase (HAp) in the crystal forme at the surface of the collagen membrane. HAp powder was further used to obtain a hybrid mixture with CHI (in accordance with the section Materials). The most intense peaks of HAp powder (O) are at 31.8°, 32.2°, 32.9°, 25.9°, and 49.5° (Figure 1a). In accordance with the literature [25] and our previous studies [26], the HAp diffractogram obtained this way indicates a crystalline form. The collagen–CHI–HApNP diffractogram confirms the presence of HAp and CHI, as well. CHI is defined by the diffractogram with two clearly marked peaks in the positions 10.2° (*) and 19.8° (*), which was defined in our earlier research [26]. The peaks originating from CHI were not sharp. The synthesized HAp powder consists of individual particles with a nanorod-type morphology. Morphology of the surface of synthesized HAp powder and the surface of modified membrane were visualized with SEM. All particles (Figure 1b) had an average diameter of 15–30 nm and a length of around 60–100 nm. The HAp particles (marked with arrows) are distributed as individuals but also as agglomerated in the matrix of CHI (Figure 1c) on the surface of the membrane.

**Figure 2:**
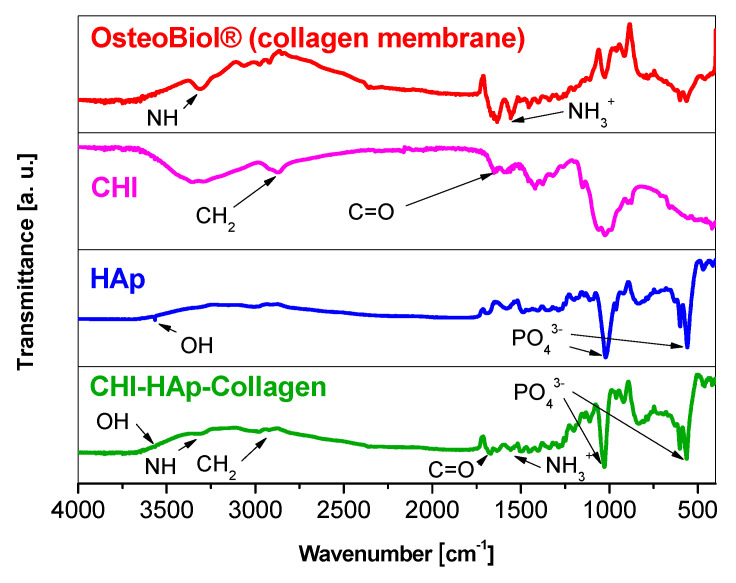
ATR FTIR spectra of OsteoBiol (collagen membrane), chitosan (CHI), nano-hydroxyapatite (HApNPs), and CHI–HAp–collagen.

The FTIR spectra of the collagen membrane, CHI, HApNPs, and CHI–HAp–collagen are in the frequency range from 4000 to 400 cm^−1^ (Figure 2). FTIR analysis confirmed results from XRD, indicating the presence of CHI and HAp phase at the surface of collagen membrane. Absorption bands at about 3310 cm^−1^ originate most probably from the stretching vibration of the NH group of the secondary amines from the collagen membrane. An absorption band appears at about 1550 cm^−1^ arising from the symmetric NH^3+^ bending vibrations from collagen, and also [41], the FTIR spectra of CH display characteristic peaks at 2880 cm^−1^, which is assigned to the CH_2_ stretching vibration, and 1650 cm^−1^, which is due to the C=O vibration in the amide group (amide I band) [42]. The adsorption band at around 560 cm^−1^ could be ascribed to the bending mode of PO_4_^3-^ from HAp. The adsorption band at around 560 cm^−1^ could be ascribed to the bending mode of PO_4_^3-^ from HApNPs. The band at 1027 cm^−1^ could be assigned to the stretching vibration of PO_4_^3-^, as well as from HApNPs. The obtained results of the FTIR analysis are consistent with our previous results and the results of other authors [43,44]. The presence of the characteristic bands of the membrane, CHI, and HAp in CHI–HAp–collagen suggest that CHI and HAp were successfully decorated onto the surface of membrane.

### 3.2. DPSCs Viability

**Figure 3:**
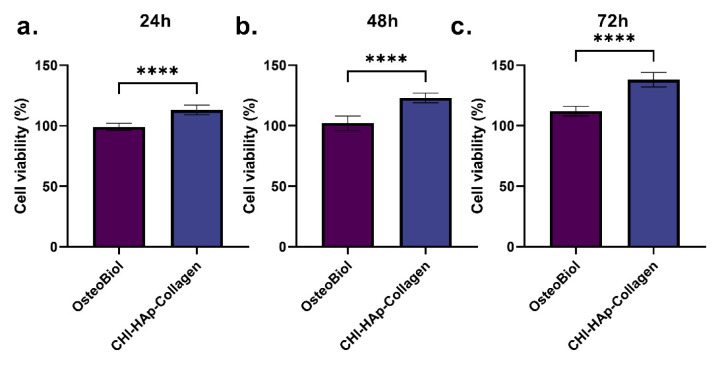
DPSC viability after (**a**) 24, (**b**) 48, and (**c**) 72 h of treatment. OsteoBiol—collagen membrane; CHI–HAp–collagen—collagen membrane with addition of nano-hydroxyapatite and chitosan; **** *p* < 0.0001.

The viability of DPSCs cultured for 24, 48, and 72 h is depicted in Figure 3. Neither membrane type was cytotoxic to DPSCs. In addition, at all times, the cell viability on CHI–HAp–collagen was significantly (*p* < 0.0001) greater than those of the control membranes (without CHI and HApNPs), indicating a higher proliferation rate of the cells seeded on CHI–HAp–collagen.

### 3.3. ALP Activity

**Figure 4:**
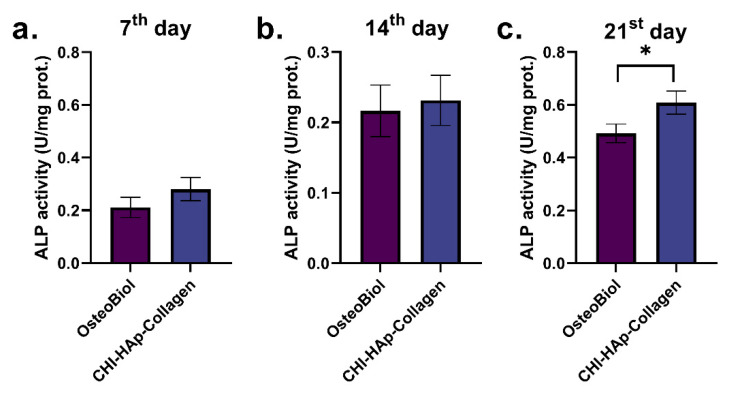
ALP activity of DPCSs during (**a**) seven, (**b**) fourteen, and (**c**) twenty-one days of osteogenic differentiation. OsteoBiol—collagen membrane; CHI–HAp–collagen—collagen membrane with addition of nano-hydroxyapatite and chitosan; * *p* < 0.05.

After 7, 14, and 21 days of DPSC osteogenic induction with membranes, the activity of the alkaline phosphatase enzyme was analyzed (Figure 4). The ALP activity of DPSCs cultured on CHI–HAp–collagen increased with time, with a significant (*p* < 0.05) difference on the 21st day after induction.

### 3.4. Osteogenic-Related Gene Expression

**Figure 5:**
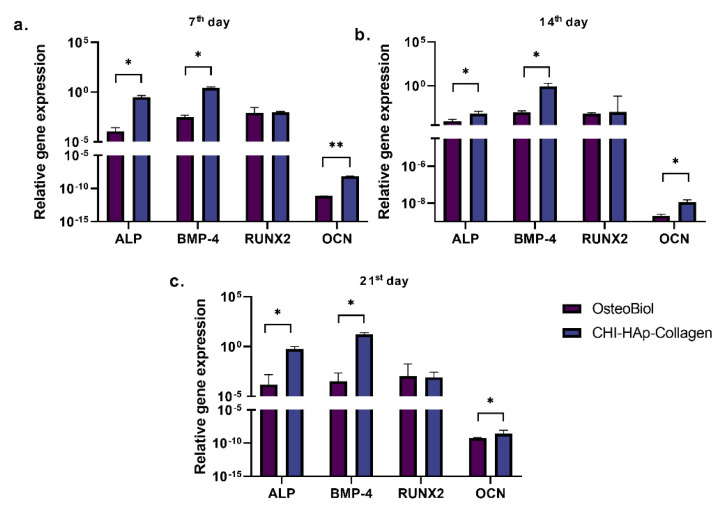
Relative expression of ALP, BMP-4, RUNX-2, and OCN genes during (**a**) seven, (**b**) fourteen and (**c**) twenty-one days of osteogenic differentiation. OsteoBiol—collagen membrane; CHI–HAp–collagen—collagen membrane with addition of nano-hydroxyapatite and chitosan; * *p* < 0.05, ** *p* < 0.01.

After 7, 14, and 21 days of osteogenic induction, the expressions of the osteogenic-related genes ALP, BMP-4, RUNX2, and OCN were evaluated. At each time point, the expression of ALP, BMP-4, and OCN significantly increased in DPSCs grown in the presence of CHI–HAp–collagen. DPSCs seeded on the CHI–HAp–collagen membrane exhibited no statistically significant difference in RUNX2 gene expression compared to those seeded on the unmodified membrane (Figure 5).

### 3.5. Colony-Forming Units on Membranes and in Medium around Membranes and SEM Analysis

**Figure 6:**
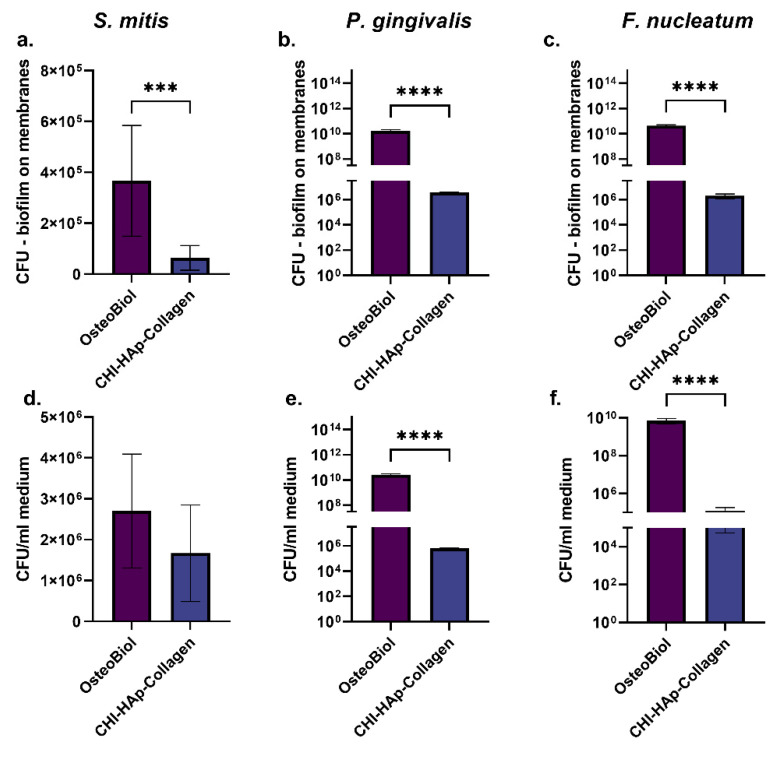
CFUs of monomicrobial biofilm of *S. mitis*, *P. gingivalis*, and *F. nucleatum* (**a**–**c**) formed on different collagen membranes and CFUs (**d**–**f**) in medium around the membranes. OsteoBiol—collagen membrane; CHI–HAp–collagen—collagen membrane with addition of nano-hydroxyapatite and chitosan; *** *p* < 0.001, **** *p* < 0.0001.

The results of the quantification of three different monomicrobial biofilms formed on both types of collagen membranes showed significantly higher values of CFU on control membranes compared to CHI–HAp–collagen membranes for each monomicrobial biofilm (Figure 6). Additionally, the CFU count from the medium around both types of collagen membranes was higher for the medium around unmodified membranes for all species (results for *P. gingivalis* and *F. nucleatum* reached statistical significance).

**Figure 7:**
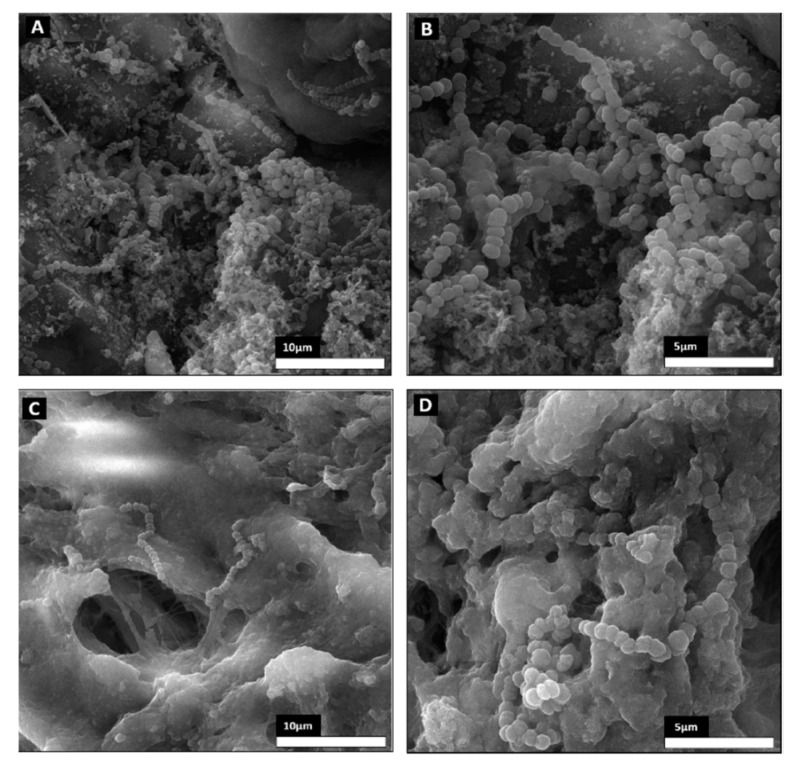
SEM images of *S. mitis* biofilm on the surfaces of (**A**,**B**) control collagen membranes and (**C**,**D**) CHI–HAp–collagen membranes. Scale bars: 5 μm and 10 μm, magnifications 10,000× and 5000×, respectively.

A monomicrobial biofilm of *Streptococcus mitis*, as the primary colonizer, was cultured on both control and CHI–HAp–collagen membranes and was visualized by SEM. Images of the control membranes showed the presence of monomicrobial biofilms across the surface of the membrane, consisting of clusters of microorganisms embedded in the extracellular matrix (Figure 7). On the surface of the CHI–HAp–collagen membranes, fewer chain-organized microorganisms were found, with little or no presence of extracellular matrix.

## 4. Discussion

In this study, the most widely used biomaterial for GBR, collagen membrane, was decorated with nano-hydroxyapatite nanoparticles (HApNPs) and low-molecular-weight chitosan (CHI) to enhance its antibacterial and osteoinductive properties. The XRD pattern and scanning electron microscopy of the synthesized hydroxyapatite powder indicated that a crystalline form was obtained and that the particles were less than 100 nm in diameter. The XRD patterns of collagen membrane and CHI display no sharp peaks originating from the membrane (collagen) or CHI because these polymers are mostly amorphous, which is in accordance with XRD studies from our previous work and in other authors’ publications [43,45,46]. The most intense peaks (O) around at 2θ = 31.8, 32.9, 25.9, and 46.7° originate from calcium hydroxyapatite (HAp) in accordance with JCPDS File No. 9-432, International Center for Diffraction Data. The diffractogram also shows peaks (+) at 2θ = 27.8 and 31°, which most likely originate from tricalcium phosphate (TCP). The infrared spectroscopy analysis of the modified collagen membrane confirmed that membranes were successfully decorated with HApNPs and CHI. FTIR spectra confirmed the existence of CHI and HAp on the collagen substrate (membrane). The band observed at 3564 cm^−1^, which originates from the stretching of the structural OH- from HAp (Figure 2), is also present. The shift to lower values of 3560 cm^−1^ in CHI–HAp–collagen could indicate the formation of a hydrogen bond between HAp and CHI. The capability of this decorated membrane to induce cell proliferation and osteoblastic differentiation of the DPSCs was assessed in this study. The results proved that decorated membranes, aside from acting as a physical barrier, also have the potential to induce bone formation. Cell viability assessments are important because they evaluate in vitro the interaction between cells and the material’s surface as the first step in evaluating the material’s biocompatibility [31]. The MTT assay demonstrated the presence of viable cells at each time point on both membranes. However, DPSCs seeded on the CHI–HAp–collagen membrane showed higher proliferation rates than those in the control group. These findings suggest that a membrane surface coated with CHI and HApNPs promotes strong cell adhesion and metabolic activity. Thus, we hypothesized that CHI coupled with HApNPs can increase the survivability of cultured cells connected to these collagen membranes. These results were in line with previous studies that employed CHI in conjunction with hydroxyapatite and demonstrated excellent biocompatibility [47,48,49]. Regarding the bioactivity of CHI–HAp–collagen, the results showed that the ALP activity of DPSCs seeded on the CHI–HAp–collagen membrane was stimulated more than that of those seeded on membranes without these decorations, despite the fact that the difference was significant just after 21 days of culture. The bioactivity of both membranes was also assessed for the expression of osteogenesis-related genes in DPSCs. A significant increase in the expression of ALP, BMP-4, and OCN was observed at each time point, mainly at the mature stage of cells. In particular, the secretion of osteocalcin is linked to alkaline phosphatase activity, and both the ALP and OCN genes are osteoblastic markers in the control of fundamental functions in the process of bone remodelling [50]. BMP-4 mRNA levels were also increased by CHI–HAp–collagen. BMP-4 is a member of the transforming growth factor superfamily that is important in tooth generation and formation, osteoblast differentiation, and matrix secretion [51]. On the other hand, the level of RUNX2 mRNA was similar in DPSCs seeded on both membranes. Other research has found that the Runt family protein RUNX2 is typically expressed during the early differentiation period in hDPSCs and peaks on day 5, indicating that RUNX2 may influence the direction of these stem cells towards odontoblastic differentiation [52,53]. Given that RUNX2 was not enhanced while ALP, OCN, and BMP-4 expression were up-regulated in CHI–HAp–collagen compared to unmodified membranes, this demonstrated that CHI and HApNPs can stimulate not only cell proliferation but also the differentiation of DPSCs in the osteoblast lineage.

It has been well known that bacterial infections can hamper osteogenesis as well as bone repair [53]. CHI has been shown in studies to affect the permeability of bacterial membranes. The antimicrobial action may be achieved by the binding of positively charged amino groups of CHI to the cell wall polymers or cytoplasmic membrane of a bacterial cell, activating some effects, such as the disruption of bacterial membrane function, leading to a change in the permeability and efflux of ions and proteins from the cytoplasm to the extracellular space [54,55,56,57,58]. Therefore, we hypothesized that the dual activity of being both antibacterial and osteogenic can be simultaneously achieved by using HApNP/CHI decorations. A strong and significant antibiofilm activity was observed for the CHI–HAp–collagen membrane for all tested bacterial strains by counting the live bacteria from the biofilm. The results also showed a decrease in the number of bacteria in the medium around the CHI–HAp–collagen membrane. The number of CFUs of Gram-negative *P. gingivalis* and *F. nucleatum* in the medium around the decorated membranes was significantly lower, indicating the release of antimicrobial substances and an antimicrobial effect around the membrane. The number of CFUs of Gram-positive *S. mitis* in the medium around the CHI–HAp–collagen membranes also decreased but without statistical difference. Previous studies showed the antibacterial effect of different types of CHI on both bacteria and fungi [56,59,60]. Some studies reported better effects against Gram-positive microorganisms [61], while others reported better effects against Gram-negative microorganisms [62]. The molecular weight of CHI is one of the most important variables influencing these effects. The CHI used in this study has a low molecular weight, and a number of studies have demonstrated that CHI with a low molecular weight is more effective against Gram-negative bacteria, as demonstrated in our study [63,64].

Oral streptococci, as the quantitatively most prevalent microorganisms in the oral environment, are thought to be the early colonizers of oral surfaces. As a substrate for the bacterial adhesion of other microorganisms, their role is crucial in the process of polymicrobial biofilm formation [65]. The further attachment of *F. nucleatum*, known as a “bridge” microorganism, enables the attachment of late colonizers, such as *P. gingivalis*, commonly related to the pathogenesis of periodontal diseases [66]. Thus, in this study, we evaluated whether the surface of control and decorated collagen membranes are susceptible to *S. mitis* adhesion both by counting CFUs and visualization with SEM. Even if SEM analysis does not distinguish live from dead bacteria cells, the formation of a mature biofilm characterized by an extracellular matrix can be observed. SEM images of control membranes showed the presence of an extracellular matrix around microorganisms, which is a characteristic of a mature biofilm. On the other hand, a decorated membrane contained fewer microorganisms and did not form a biofilm structure.

Overall, this study demonstrated that CHI–HApNP-coated membranes with combined antibacterial and biological activities have great potential for use in bone regeneration applications. However, the optimization of the decoration, the rate of degradation, and the long-term antibacterial effect of the CHI–HAp–collagen membrane should be thoroughly investigated in future studies.

## 5. Conclusions

In conclusion, the results of the MTT assay indicate that there is no cytotoxicity in either the control membranes or membranes decorated with CHI–HApNPs. The results of the ALP activity assay and qPCR for BMP4, ALP, RUNX2, and OCN show that there is an increase in osteogenic activity on decorated membranes. An antibiofilm effect was demonstrated on decorated membranes for all tested bacterial species. An antimicrobial effect on the surrounding medium was demonstrated for the tested Gram-negative bacteria, *F. nucleatum* and *P. gingivalis.*

## Figures and Tables

**Table 1:** Primers with corresponding sequences used in the study.

Product Name		Sequences (5′→3′)
ALP	Forward Reverse	CCACGTCTTCACATTTGGTG ATGGCAGTGAAGGGCTTCTT
BMP2	Forward Reverse	CACTGTGCGCAGCTTCC CCTCCGTGGGGATAGAACTT
OCN	Forward Reverse	TTGGACACAAAGGCTGCAC CTCACACTCCTCGCCCTATT
RUNX2	Forward Reverse	ACAAACAACCACAGAACCACAAGT GTCTCGGTGGCTGGTAGTGA
GAPDH	Forward Reverse	TCATGACCACAGTCCATGCCATCA CCCTGTTGCTGTAGCCAAATTCGT

**Table 2:** Growing conditions of reference strains: *Porphyromonas gingivalis* ATCC 33277, *Fusobacterium nucleatum* ATCC 25586, and *Streptococcus mitis* ATCC 13770.

Reference Strain	Medium	Agar	Temperature	Incubation Time	Conditions
*Porphyromonas gingivalis*	Schaedler broth with hemin and vitamin K1 *	Brucella agar with 5% sheep blood ***	37 °C	5 days	Anaerobic
*Fusobacterium nucleatum*	Schaedler broth with hemin and vitamin K1*	Brucella agar with 5% sheep blood ***	37 °C	5 days	Anaerobic
*Streptococcus mitis*	Brain heart infusion (BHI) broth**	Columbia agar with 5% sheep blood ****	37 °C	48 h	Anaerobic

Manufacturers of growth medium: * Becton, Dickinson and Co., Franklin Lakes, New Jersey. Ltd., USA, ** HiMedia, India, *** Sigma Aldrich, MO, USA, **** ProReady (Belgrade, Serbia).

## Data Availability

Not applicable.
